# Social Support Mechanisms in an Online Type 1 Diabetes Community: Social Network Analysis of Stakeholder Diversity and Disease Duration

**DOI:** 10.2196/82996

**Published:** 2026-06-15

**Authors:** Yujia Zhu, Yanxiang Zhang, Chenxiao Zhao, Xifeng Zeng, Xueying Zheng

**Affiliations:** 1 Department of Endocrinology and Metabolism, The First Affiliated Hospital of USTC, Division of Life Sciences and Medicine, University of Science and Technology of China, No. 5 Building, South Campus (Anhui Provincial Hospital), Hefei, Anhui, 230036, China, 86 13750400548; 2 Department of Communication of Science and Technology, University of Science and Technology of China, Hefei, Anhui, China; 3 Shenzhen Aibowei Biotechnology Co., Ltd. (Tangtangquan), Shenzhen, Guangdong, China

**Keywords:** diabetes mellitus type 1, social support, online health communities, social network analysis, large language models, sentiment analysis, peer group

## Abstract

**Background:**

Online health communities (OHCs) have emerged as critical platforms for patients with type 1 diabetes (T1D) to exchange informational and emotional support. However, how stakeholder roles and disease duration jointly shape support dynamics and influence formation remains underexplored.

**Objective:**

This study aimed to examine network-based social support mechanisms in a large T1D OHC, focusing on how stakeholder diversity and disease duration are associated with social support behaviors, subnetwork structures, and user influence.

**Methods:**

This retrospective observational study analyzed digital trace data from China’s largest T1D online community (January 1-May 20, 2024), comprising 43,788 posts and 145,423 comments contributed by 1393 users. We manually annotated 2000 randomly sampled posts and fine-tuned a GPT-4o-mini (OpenAI) to classify support type (informational or emotional, and seeking or providing), yielding 20,384 support-related posts and 56,953 comments from 1224 users. We constructed weighted directed informational and emotional interaction networks and modeled predictors of a composite influence metric (Relative Centrality) using a gamma log-link generalized linear model (including demographics, identity, sentiment, disease duration, posting orientation, and cyclical activity time). Analyses were conducted in Python (version 3.11; Python Software Foundation). Statistical significance was set at *P*<.05.

**Results:**

Support predominantly flowed from longer-duration members (≥ y) to those at earlier stages (≤5 y). Both subnetworks exhibited multicentered, star-like structures; the informational subnetwork had broader participation (density 0.031, diameter 7), while the emotional network was denser (density 0.039, diameter 6). In the influence model, peer supporters had substantially higher influence than patients (exp(β)=34.79, 95% CI 18.94‐64.08; *P*<.001), professionals lower (exp(β)=0.41, 95% CI 0.17‐0.99; *P*=.055), and women higher than men (exp(β)=1.65, 95% CI 1.23‐2.23; *P*=.001). Positive sentiment was associated with higher influence (exp(β)=1.91, 95% CI 1.22‐2.97; *P*=.005), and negative lower (exp(β)=0.54, 95% CI 0.37‐0.79; *P*=.001). Influence followed an inverted U-shaped trajectory over disease duration, peaking at approximately the 116th month (95% CI 43.25‐188.91).

**Conclusions:**

This study suggests that social support patterns and user influence in a T1D OHC vary by stakeholder role and disease duration. Users with shorter disease duration more often sought support, whereas longer-duration users more often provided support, and informational and emotional exchanges formed distinct interaction subnetworks. Peer supporters were the most influential users; influence was also associated with gender, sentiment, activity timing, and a nonlinear (inverted U-shaped) relationship with disease duration. These findings may inform peer-facilitated, stage-tailored community strategies, with professionals engaged in targeted, complementary roles. A patient-centered collaborative care approach integrating peer experience with multidisciplinary clinical input could be explored in future work.

## Introduction

### Background

Type 1 diabetes (T1D) commonly develops during adolescence but can occur at any age [1]. As there is currently no cure, individuals diagnosed with T1D require lifelong management. Because clinical characteristics, complication risks, and mortality are closely associated with disease duration [2,3], the long-term demands of intensive self-management and cumulative complication risk may increase patients’ psychosocial burden, potentially contributing to emotional distress, behavioral difficulties, and poor treatment adherence [4]. As of 2024, an estimated 599,000 individuals are living with T1D in China, accounting for approximately 6.5% of the global total [5]. Although the incidence of T1D in China remains relatively low, the large population size results in one of the highest national case counts worldwide [1]. The primary disease burden among Chinese individuals with T1D includes poor glycemic control, high rates of complications, and considerable financial strain [6]. Understanding long-term management and support mechanisms for T1D in China is particularly important, given the uneven access to offline social support and the growing role of digital health platforms in providing continuous care.

T1D management primarily occurs outside clinical settings. Due to geographic dispersion, many individuals with T1D struggle to connect with peers who share similar medical experiences [7], making family members and friends their primary sources of support. In contrast, online health communities (OHCs) provide more accessible social environments that facilitate peer interaction and experience sharing, and thus serve as critical platforms for social support exchange [8]. Studies have shown that when individuals share their disease trajectories, treatment outcomes, and coping strategies in OHCs, it facilitates the exchange of diabetes-related knowledge and health information, which can substantially enhance disease management. Beyond peer interaction, OHCs also serve as venues where health care professionals and other stakeholders can engage individuals with T1D. For example, physicians and nurses who actively participate in online discussions can bridge medical expertise with lived experiences, fostering trust, promoting adherence, and ultimately improving health outcomes [9]. Despite growing interest in online social support, existing OHC-based research has paid limited attention to the co-evolution of social support behaviors and social network structures in long-term T1D management. As a result, identifying and empowering key actors in support networks, along with developing sustainable T1D-specific online social support systems, remains a critical challenge for advancing targeted and durable health communication and intervention.

### Social Support From a Diverse Stakeholder Perspective

Social support refers to information that leads individuals to perceive themselves as cared for, loved, respected, and integrated into a mutual aid network [10]. It is typically categorized into informational, emotional, instrumental, and appraisal support [11,12]. Among these, informational and emotional support are the most prevalent forms on social media, playing a crucial role in facilitating health decision-making and improving health outcomes [13,14]. In online communities, informational support is typically sought through question asking and advice seeking, whereas emotional support is expressed through sharing illness experiences, emotional reactions, and coping narratives [15,16]. The type and emotional tone of posts influence the likelihood of receiving support, with emotionally expressive negative posts more likely to elicit supportive responses [17,18]. Moreover, even users who do not actively participate in discussions can benefit indirectly from observing others’ emotional exchanges, thereby gaining valuable informational resources in the process [19]. These patterns highlight the heterogeneity of support-seeking behaviors in T1D online communities and suggest that variation in support sources is a key factor in understanding how social support functions [14,20].

Contemporary research on online social support in T1D follows 2 primary trajectories. The first centers on professional support offered by health care providers, emphasizing medically guided knowledge and structured intervention. Health care professionals are among the most reliable sources of informational support [21], with numerous studies showing that such information is typically more accurate, evidence-based, and trusted by individuals with T1D [22]. As a sustainable resource, professional informational support complements the emotional support more commonly exchanged among peers, jointly promoting improved self-care and behavioral outcomes [22]. The second line of research focuses on experiential support, exchanged among patients and caregivers through shared experiences and mutual understanding. According to the principle of network homophily, individuals are more likely to trust and adopt advice from those with whom they share similar health trajectories [23,24]. Unlike professionally sourced support, peer-generated content tends to be more personalized, emotionally resonant, and reflective of day-to-day management challenges [25]. This type of experiential knowledge, accumulated through lived experience and long-term observation of disease progression, positions patients and caregivers as potential lay experts in the T1D community [26]. Taken together, these 2 trajectories underscore the need to understand how professional and experiential support interact within broader community networks, rather than in isolation.

Gender is a salient factor influencing patterns of seeking and providing social support in online communities, with implications for health outcomes [27]. In terms of posting motivation, women are generally more active in maintaining relationships through written interactions and post more frequently than men [28]. At the content level, men’s posts are more technical and information-focused, often using medical terminology, whereas women are more likely to seek emotional support and express subjective concerns or negative emotions [8,29]. Women also report receiving more emotional support from social networks, while men more often rely on their partners as their primary source [30]. These gendered tendencies shape both the type of support exchanged and the emergent network configuration, providing context for subsequent analyses of stakeholder roles.

Beyond individual attributes, stakeholder interactions in T1D communities involve diverse and often asymmetrical support dynamics. The psychosocial burden of T1D, particularly its impact on quality of life, extends beyond individuals with T1D to caregivers and family members [31], shaping how different groups engage and what types of support they prioritize. Yet, most studies remain focused on dyadic support exchanges, neglecting the broader collaborative mechanisms across heterogeneous actors. Understanding these complex, multistakeholder patterns is critical to designing more effective and sustainable support systems in OHCs.

### Social Support From the Perspective of Disease Duration

Following diagnosis, individuals with T1D undergo a prolonged adaptation process during which disease characteristics, complication risks, and mortality vary by age and disease duration [2,3]. Many also experience diabetes-related stigma, contributing to emotional and psychological distress [32]. Previous research highlights the temporal dynamics of social support in OHCs. Informational support often fulfills immediate needs, while emotional support fosters long-term engagement. Previous work by Wang et al [33] suggests that informational support is effective for addressing short-term challenges, whereas emotional support strengthens interpersonal ties, promoting long-term engagement in support groups [34]. These temporal patterns indicate that support needs evolve over the disease duration, underscoring the importance of timing in community-based interventions.

Support behaviors in type 2 diabetes communities shift with disease duration; prediabetic users typically seek information, while insulin-treated users more frequently provide both informational and emotional support [35]. Similar patterns are observed in T1D communities. Wang et al [36] found that newcomers primarily sought emotional support, but gradually shifted toward providing informational support as their engagement deepened. Patients and caregivers tend to seek more external support during early diagnosis to cope with uncertainty and emotional distress [13]. As experience accumulates, reliance on external information often decreases [37]. Accordingly, it has been recommended that self-management of education and support for T1D be tailored to key time points, such as diagnosis, unmet treatment goals, psychosocial or physiological transitions, and major changes in care [38]. However, little is known about how support behaviors evolve across stakeholder groups across the disease course, leaving a critical gap in understanding the sustainability of online social support systems. This gap motivates our first research question (RQ) 1.

### Social Support Networks Within Diabetes OHCs

In public health, social connection is a key determinant of effective health management and long-term behavioral change [39], with perceived social support widely used as a proxy for tie strength. Social network analysis (SNA) offers a systematic approach to mapping interaction structures, identifying influential actors, and understanding network formation and evolution. Previous studies show that individuals occupying central positions in diabetes-related networks play a greater role in information diffusion [40]. Gender also shapes network structure. Although men typically maintain smaller social networks [41], they often occupy more central positions [8]. Disease duration is another factor. Dias et al [42] found that users diagnosed for less than 2 years tended to be more active, whereas those with longer disease duration (≥2 y) were more likely to occupy central positions, suggesting a potential association between disease duration and network centrality. This evidence motivates the second RQ, which examines how stakeholder interactions shape informational and emotional subnetworks, and informs hypotheses about influence formation.

In recent years, researchers have increasingly applied SNA to examine support networks among T1D stakeholders. Earlier work primarily focused on professional medical networks, emphasizing the roles of health care providers in information exchange and care coordination [43]. In contrast, Wu et al [19] adopted an experimental design to construct a Facebook (Meta)-based virtual peer network consisting of 212 individuals with T1D, in which 10 highly active participants were trained as peer leaders. These leaders exhibited centrality scores nearly 10 times higher than regular members, positioning them as influential brokers within the network. Their high betweenness centrality allowed them to bridge subgroups and facilitate diffusion of health behaviors and information [44-46]. Identifying such high-influence nodes is therefore critical to enhancing the effectiveness and sustainability of online T1D self-management systems [47], which motivates the third RQ3.

Limited attention has been paid to the mechanisms by which informational and emotional support circulate across diverse stakeholder groups over the course of T1D. Using data from China’s largest T1D OHC, we analyzed user-generated posts and comments. A manually annotated subset of posts was used to fine-tune a large language model (LLM) for automated classification of social support types, followed by SNA to map interaction networks and generalized linear model (GLM) regression to examine factors associated with user influence. Drawing on social support theory, this research integrates stakeholder diversity and disease duration to examine who seeks and provides support across stakeholder groups, how these behaviors evolve with disease duration, and through what mechanisms influence forms among support participants. The findings aim to provide empirical evidence to inform personalized health interventions and the development of sustainable online social support systems, ultimately supporting improved health management for patients with T1D. Accordingly, we pose the following RQs:

- RQ1: What are the characteristics of social support interactions among diverse stakeholders in T1D online communities, and how do these interactions evolve across different stages of disease duration?
- RQ2: How do stakeholder interactions collectively shape the structural characteristics of informational and emotional support subnetworks?
- RQ3: Which stakeholder groups hold the highest influence in T1D online communities, and what mechanisms drive the formation of their influence?

## Methods

### Data Collection and Cleaning

We analyzed user-generated digital traces from the largest online T1D community in China, covering the period from January 1 to May 20, 2024. The dataset comprised 43,788 posts, 145,423 top-level comments, and profiles for 1393 users. Metadata included post and comment time stamps, user identifiers (ID and nickname), registration dates, gender, community identity, and time since diagnosis.

Before analysis, we preprocessed the post texts following methods outlined by Kumar et al [48]. First, irrelevant content such as “@username,” “#Topic,” and URLs were removed, and duplicate entries were eliminated, resulting in a final dataset of 42,321 posts. From the cleaned dataset, a simple random sample of 2000 posts was drawn for manual annotation. The coding scheme was developed by the conceptualization of social support in networks by House et al [11], support dimensions by Cutrona and Russell [49], and typology of support types by Myrick et al [50]. A subset of 500 posts was first selected as a coding sample and independently annotated by 2 trained researchers (Y Zhu and CZ). Posts were initially classified as either related or unrelated to social support. Those deemed relevant were then further categorized as informational or emotional in content, and finally distinguished as either seeking or providing support (Cohen κ=0.713). An additional 500 posts were subsequently coded to refine and validate the coding scheme, ensuring clarity, reproducibility, and mutually exclusive categories (Cohen κ=0.776). Detailed operationalizations of the key terms used in this study are provided in Multimedia Appendix 1. The remaining 1000 posts were then annotated by the coders, yielding a manually labeled dataset of 2000 posts in total (Cohen κ=0.802).

We fine-tuned the GPT-4o-mini (OpenAI) model on the manually labeled dataset, using 75% of the data for training and 25% for validation. The model was trained over 3 epochs with a batch size of 16. The full validation loss across the entire validation set was 0.073. In addition, the model achieved a validation accuracy of 0.977. For a rigorous and unbiased performance evaluation, we conducted an independent inference test on fresh model weights, achieving an accuracy of 0.878 with a macro *F*_1_-score of 0.869, indicating acceptable generalization performance and reliable classification of relevant posts. After removing unrelated posts, the dataset retained 20,384 posts containing either informational or emotional support, along with 89,352 corresponding comments. After further cleaning, which involved removing irrelevant content, emojis, and self-replies from the comments, a final set of 56,953 valid comments and data from 1224 users were obtained.

### Ethical Considerations

All personally identifiable information was anonymized during preprocessing. Ethical approval for this study was granted by the Clinical Trial Ethics Committee of Anhui Provincial Hospital (review 2019KY-Lunshen-27). No compensation was provided because no participants were recruited or contacted.

### Sentiment Analysis

Sentiment analysis (SA), a text mining technique, is designed to extract individual opinions and detect the polarity and intensity of emotions expressed in textual data [51]. In this study, the sentiment knowledge enhanced pretraining model was used to perform sentence-level sentiment classification. This approach enables the extraction of fine-grained semantic features and improves classification accuracy [52,53]. For each post and comment, we implemented a binary classification task in which the model outputs a sentiment label (positive or negative) along with a confidence score ranging from 0 to 1. Higher scores indicate greater confidence in the predicted sentiment class. To ensure comparability across all samples, we defined a normalized sentiment score (SentiScore; Multimedia Appendix 2).

To ensure the reliability and accuracy of sentiment classification, 1721 posts related to informational and emotional support were selected from a manually labeled dataset of 2000 posts. These posts were independently annotated using a binary sentiment classification scheme by 2 trained researchers (Y Zhu and CZ), achieving a high interrater agreement (Cohen κ=0.814), indicating the dataset’s suitability for the study context. To evaluate model performance, 75% of the dataset was used for training and 25% for testing. On the test set, the model achieved an accuracy of 94.7%, comparable with its 95.4% accuracy on the Chinese SA dataset ChnSentiCorp [53]. The model also achieved a macro *F*_1_-score of 0.943, mitigating concerns related to class imbalance [54]. Additionally, precision and recall were 96.6% and 94.8%, respectively, with an area under the receiver operating characteristic curve of 94.6%. These results demonstrate that the sentiment knowledge enhanced pretraining model performs robustly in SA within the T1D context and effectively identifies users’ emotional expressions in posts.

### SNA

In social networks, user interactions are typically conceptualized as the formation of links. Users who participate in discussion threads are treated as nodes, and interactions between them form the edges of the network. Subnetworks represent specific subsets within the overall structure [55]. Drawing on users’ posting and commenting behaviors in the T1D online community, we constructed a weighted directed network *G*=(*V*, *E*). A directed edge (*u*, *v*) indicates that *u* commented on a post by *v*, with edge weights determined by the number of comments exchanged [56]. Network density captures overall connectivity, defined as the ratio of observed directed ties to all possible ties. The diameter of the network refers to the longest shortest path between any 2 nodes, reflecting the network’s structural span and reach.

Network centrality metrics are widely used to identify influential actors in information diffusion processes [57-61]. Eigenvector centrality captures a node’s influence based not only on its direct connections but also on the importance of the nodes to which it is connected [62]. As such, ties to highly influential nodes substantially elevate a node’s status in the overall network structure. Betweenness centrality, on the other hand, captures the extent to which a node lies on the shortest paths between other pairs of nodes [58]. Nodes with high betweenness centrality often occupy key positions in information flow. Accordingly, we used the NetworkX library to compute centrality measures, weighting eigenvector centrality by comment count and betweenness centrality by its inverse. Network visualization was conducted using Gephi (Gephi Consortium) [63].

### Statistical Analysis

In the first 2 years following diagnosis, patients with T1D typically experience a marked decline in C-peptide levels, leading to severe insulin deficiency and pronounced glycemic variability [31]. The recommended time frame for screening and intervention for complications, such as retinopathy and nephropathy, is between 3 and 5 years post diagnosis [3,64]. With longer disease duration, the risk of complications associated with poor metabolic control increases. Notably, the risk of long-term complications, such as diabetic peripheral neuropathy, rises significantly after 10 years post diagnosis [65], substantially elevating patients’ mortality risk and adversely impacting both their physical and mental health. Dividing patients based on key milestones in disease duration helps better understand their social support needs, enabling targeted and individualized health interventions. In this study, users from the T1D online community were grouped by disease duration—short duration (0‐2 y and 3-5 y) and long duration (6‐10 y and 11+ y). Given the skewed distribution of posting frequency, we first used the Kruskal-Wallis test to robustly assess the main effects of disease duration and gender. Interaction effects were then examined using a 2-way ANOVA. When Levene test indicated homogeneity of variance (*P*>.05), standard *F* tests were applied; otherwise, Welch ANOVA was used to mitigate the risk of Type I error [66]. For post hoc comparisons, Tukey’s Honestly Significant Difference test was used when variance was equal, and the Games-Howell test was applied when it was not. The threshold for statistical significance was set at *P*<.05.

### Statistical Variables

To assess the impact of gender on supportive behavior, gender was coded as a binary variable (man=1, woman=0). Community identity was modeled as a 4-category variable, including patient, guardian, peer supporter, and professional, with patient serving as the reference group. The professional category includes health care providers, dietitians, mental health counselors, and diabetes education specialists; for these users, disease duration reflects years of participation in the OHC.

Demographic attributes can shape a node’s influence within a network, and an individual’s marginal influence often declines with increasing social exposure [67]. Accordingly, disease duration was modeled in 2 forms, that is, a continuous variable (months) representing time since diagnosis for patients or years of service for professionals, and a quadratic term (months^2^) to capture potential diminishing returns to network embeddedness. To control for heterogeneity in disease duration across identity groups, the variable was standardized (*z* score) within each group.

Mallipeddi et al [68] noted a positive correlation between users’ emotional tone and their engagement on social media; emotionally charged content tends to be shared more rapidly and widely than neutral content. Such interactions are often used to assess user influence [69]. Building on this, this study calculated the mean and SE of users’ SentiScore derived from their posts and comments. A *z* test (α=.001, *z*=3.291) was used to construct a 99.9% CI to determine overall sentiment orientation. Users were classified as positive if the lower bound of the interval exceeded the neutral threshold of 0.5, negative if the upper bound was below 0.5, and neutral otherwise. For users contributing no more than 1 post or comment, the raw SentiScore was used for classification in order to mitigate the impact of outliers in small samples and improve the robustness of sentiment categorization.

Previous studies have highlighted the importance of content value in shaping user influence, with popular posts significantly enhancing a user’s centrality within the network [70]. Given individual variation, users’ posting preferences may influence the mechanisms through which they gain influence. To capture this, we constructed a 2×2 normalized matrix based on post types, categorized along 2 dimensions: “seeking versus providing support” and “informational versus emotional support.” From this matrix, row and column vectors were extracted to represent 2 continuous variables, postcontent orientation (PCO) and postinteraction orientation (PIO). The principal argument (*θ*) between these vectors was calculated and linearly scaled to the (−1, 1) interval. A higher PCO indicates a stronger preference for informational support, while a higher PIO reflects a greater tendency to provide support.

Research has shown that users tend to receive more comments and shares when posting content during specific time periods (eg, morning or noon) on social media [71,72]. In this study, user activity across different time periods may vary by identity, and if key groups post during high-interaction periods, their network influence could increase. To quantify users’ activity patterns for posts and comments throughout the day, we first converted the average active time for each user into a circular mean, with values ranging from 0 to 24, and mapped it to radians. This resulted in the creation of a combined cyclical variable, cyclical activity time (Sine/Cosine), to capture their activity characteristics within the daily cycle.

To assess users’ structural influence within the T1D social support network, this study constructed a composite metric of network centrality. Eigenvector centrality is commonly used to measure global influence, as interacting with highly central nodes can enhance a user’s own network position [73]. Betweenness centrality reflects efficiency-driven interaction paths [59], aligning with the need-based behaviors observed in T1D OHCs, where users tend to connect with others who can meet their informational or emotional needs [74]. We then developed a composite metric “Relative Centrality,” which combines eigenvector centrality and betweenness centrality to identify key influencers in the T1D support network. Principal component (PC) analysis was applied to reduce the dimensionality of the 2 centrality measures [75]. The first PC had an eigenvalue of 1.68 and accounted for 83.97% of the variance, indicating that PC1 effectively captures the shared variance between the 2 metrics. Relative centrality, derived from the loading coefficients of PC1, was thus used as the core indicator of a user’s influence within the network.

A GLM was fitted with relative centrality as the dependent variable, using a gamma-distribution with a log-link function to accommodate the outcome variable’s positive skewness [76], with statistical significance set at *P*<.05. The ratio of residual deviance to degrees of freedom was 10.87 (>1), indicating potential overdispersion. All predictor variables had variance inflation factors between 1.021 and 1.663, all below 2, indicating no multicollinearity [77]. All analyses were conducted in Python (version 3.11; Python Software Foundation).

## Results

### Descriptive Statistics

In the T1D online community, patients accounted for 62.5% (765/1224) of users, followed by guardians (428/1224, 35%), professionals (24/1224, 2%), and peer supporters (7/1224, 0.6%). Women made up 61.1% (748/1224) of users, and men 38.9% (476/1224). The mean disease duration among patients was 7.8 (SD 7.0; median 6.7, IQR 2.7-11.3) years. In terms of social support interactions, the distribution of the 4 support types varied significantly across disease duration groups (*χ*²(9)=1459.8; *P*<.001). Posts providing emotional support were most common (n=8603, 42.2%), followed by informational support (n=5807, 28.5%), seeking emotional support (n=3311, 16.2%), and seeking informational support (n=2663, 13.1%).

**Figure 1.**
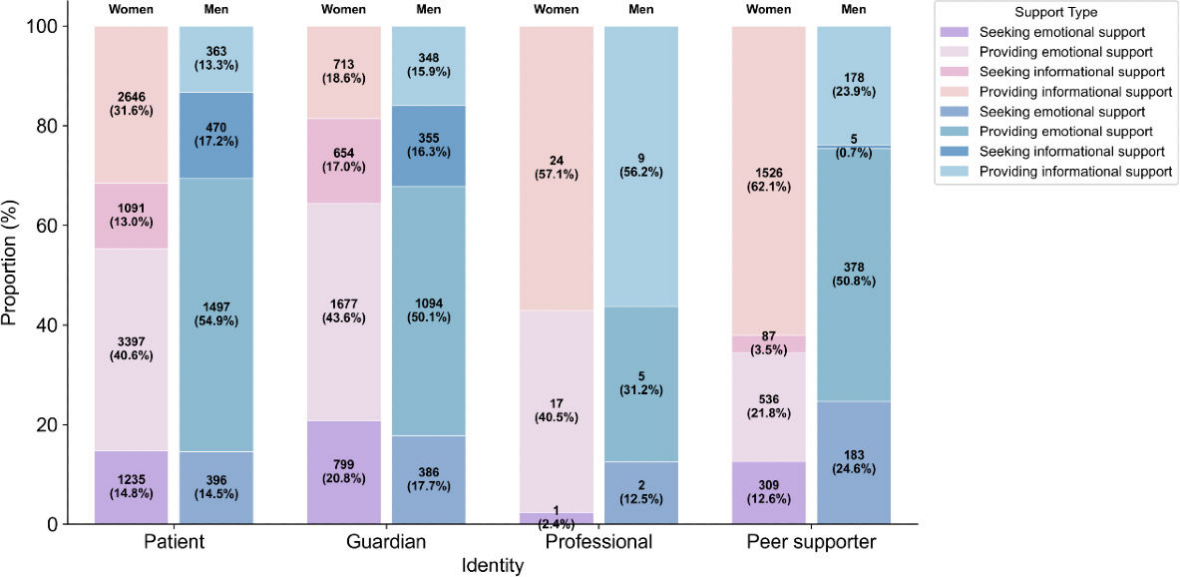
Distribution of informational and emotional support posts across stakeholder identities and genders in a retrospective observational study of China’s largest type 1 diabetes online health community (January 1-May 20, 2024).

Figure 1 illustrates the distribution of support types across stakeholder identities. Patients and guardians are the primary seekers of both informational and emotional support. Peer supporters exhibited the highest frequency of informational support provision, whereas patients contributed most frequently to emotional support. Professionals participated in both informational and emotional support interactions. Across all support types, women demonstrated higher levels of participation than men.

Kruskal-Wallis tests revealed significant differences in posting frequency by both disease duration (H=554.59, *P*<.001) and gender (H=946.51, *P*<.001), suggesting that T1D stakeholders’ engagement in social support interactions is influenced by these factors. To delve deeper into the nature of these interactions, we next examined the specific dimensions of seeking and providing support. Given significant results from Levene tests (*P*<.05), indicating heterogeneity of variance, the more robust Welch-type ANOVA was conducted to examine differences in seeking and providing support behaviors.

**Figure 2.**
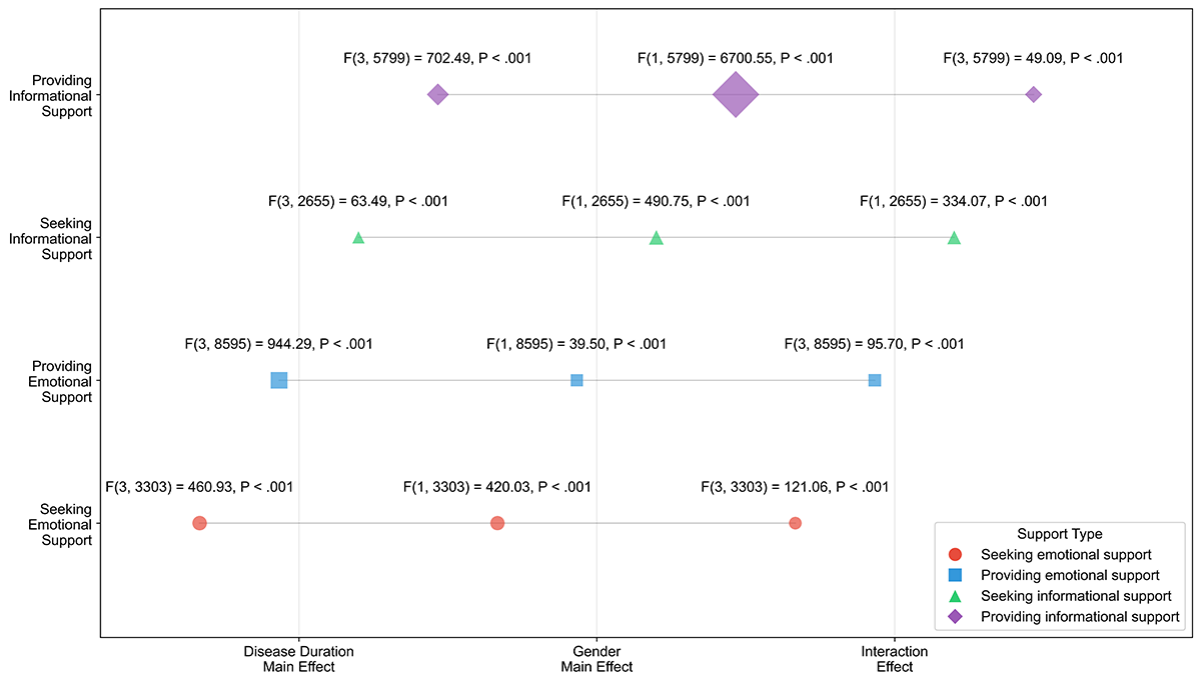
Main and interaction effects of disease duration stage and gender on the posting frequency of informational and emotional support. Results are based on a Welch-type ANOVA analyzing digital traces from a retrospective observational study of China’s largest type 1 diabetes online health community (January 1-May 20, 2024).

**Figure 3.**
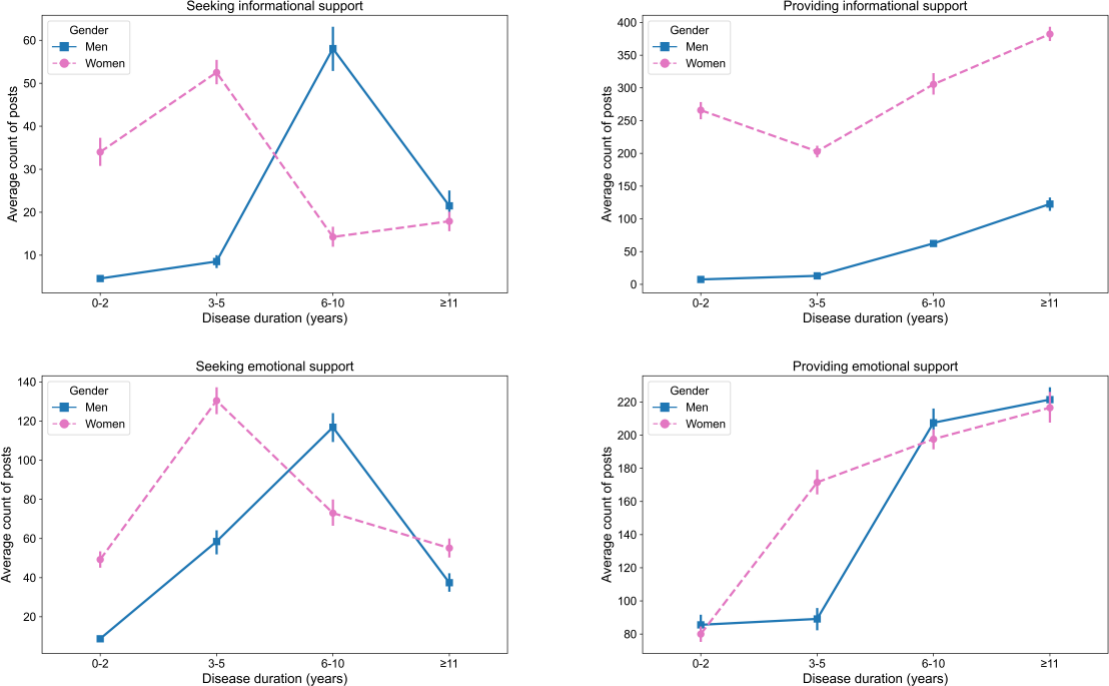
Interaction effects between disease duration stage and gender on social support behaviors (seeking vs providing, informational vs emotional) among users in China’s largest type 1 diabetes online health community (January 1-May 20, 2024).

Figure 2 indicates significant main effects and the interaction effects of both disease duration and gender on types of social support. Figure 3 illustrates the interaction effect between disease duration and gender. Overall, in the early stage (0‐2 y), users predominantly sought support, with women showing particularly high demand for emotional support. Users gradually transitioned from seeking to providing support, with provision peaking at 3‐5 years. For informational support provision, women consistently outperformed men across all disease durations. In contrast, the participation of men increased steadily with disease duration, reaching the highest frequency of informational support-seeking during the 6‐10 year stage, while the engagement of women declined after 3‐5 years. Gender differences in emotional support provision were modest, with similar activity levels between men and women in the 6‐10 year and longer duration groups.

Games-Howell post hoc tests showed that support-seeking behaviors peaked during the 3‐5 year postdiagnosis period, with higher frequencies than the 0‐2 year group for both emotional support seeking (mean 112.74, SD 94.62 vs mean 38.36, SD 40.70; *P*<.001) and informational support seeking (mean 44.00, SD 36.45 vs mean 25.72, SD 36.34; Hedges g=−0.5; *P*<.001). In contrast, support-providing behaviors increased with disease duration, particularly for emotional support provision (≥11 y: mean 218.57, SD 151.52 vs 0‐2 y: mean 82.04, SD 59.47; Hedges g=1.07; *P*<.001), while informational support provision reached its highest level at ≥11 years (mean 346.12, SD 196.20 vs mean 239.82, SD 203.12 at 0‐2 y; *P*<.001). Regarding gender differences, women provided substantially more informational support than men (women: mean 298.15, SD 200.84 vs men: mean 65.37, SD 64.40; Hedges g=1.25; *P*<.001).

### SNA

This study constructed a weighted directed network based on users’ commenting behavior in the OHC, identifying key individuals through relative centrality measures. Table 1 illustrates the distinct structures of the informational and emotional support subnetworks. The informational support subnetwork involved a broader range of users; however, its lower density indicates that these interactions were relatively diffuse. In contrast, the emotional support subnetwork was smaller in scale and had a shorter network diameter. Yet, it featured more frequent interactions and higher network density.

**Table 1.** Structural characteristics of the informational and emotional social support subnetworks.

Subnetwork	Nodes	Edges	Density	Network diameter	Average degree
Informational Support	985	29,921	0.031	7	30.377
Emotional Support	829	27,032	0.039	6	32.608

### Mapping Social Support Interactions Among Diverse Stakeholders

**Figure 4.**
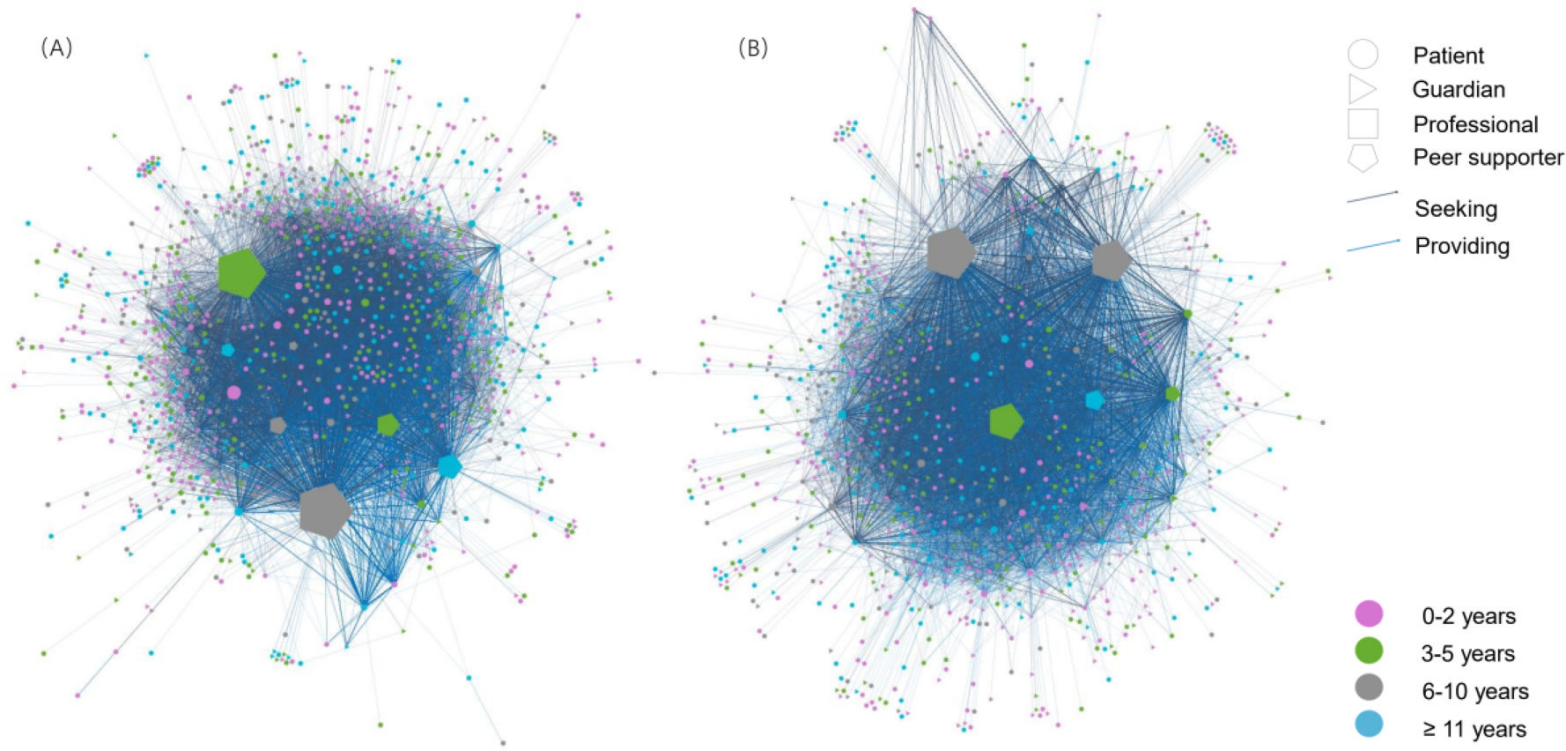
(A) Informational and (B) emotional social support interaction subnetworks from a retrospective observational study of China’s largest type 1 diabetes online health community (January 1-May 20, 2024). The networks are weighted and directed, based on comment-to-post interactions. Each node represents a user, with size indicating Relative Centrality, color representing disease duration stage, and shape denoting stakeholder identity. Arrow colors indicate the type of support (an arrow from user X to user Y indicates user X provided or sought support from user Y).

Figure 4 illustrates the social network structures of informational and emotional support interactions among diverse stakeholders. Both subnetworks exhibit a star-like topology, with multiple highly connected hubs linking to numerous peripheral nodes. Within both subnetworks, some peer supporters across different disease durations occupied central positions, forming dense local clusters and acting as key social influencers. Professionals, especially those with more than 5 years of service, were strategically placed and demonstrated high relative centrality in both networks. Some patients and guardians also formed local hubs. For example, guardians in the 0‐ to 2-year disease-duration group held influential positions in the emotional support network, while patients with ≥11 years of disease duration were central in the informational support subnetwork. In contrast, most patient and guardian nodes were sparsely connected, forming a typical core and periphery structure.

To further assess the flow of informational and emotional support among stakeholders with different disease durations, we applied Bayes theorem to infer conditional probabilities [78] and to estimate the conditional probability distributions of support-seeking and support-providing behaviors. Probabilities were normalized based on the total support interactions initiated by each disease-duration group toward other groups.

**Figure 5.**
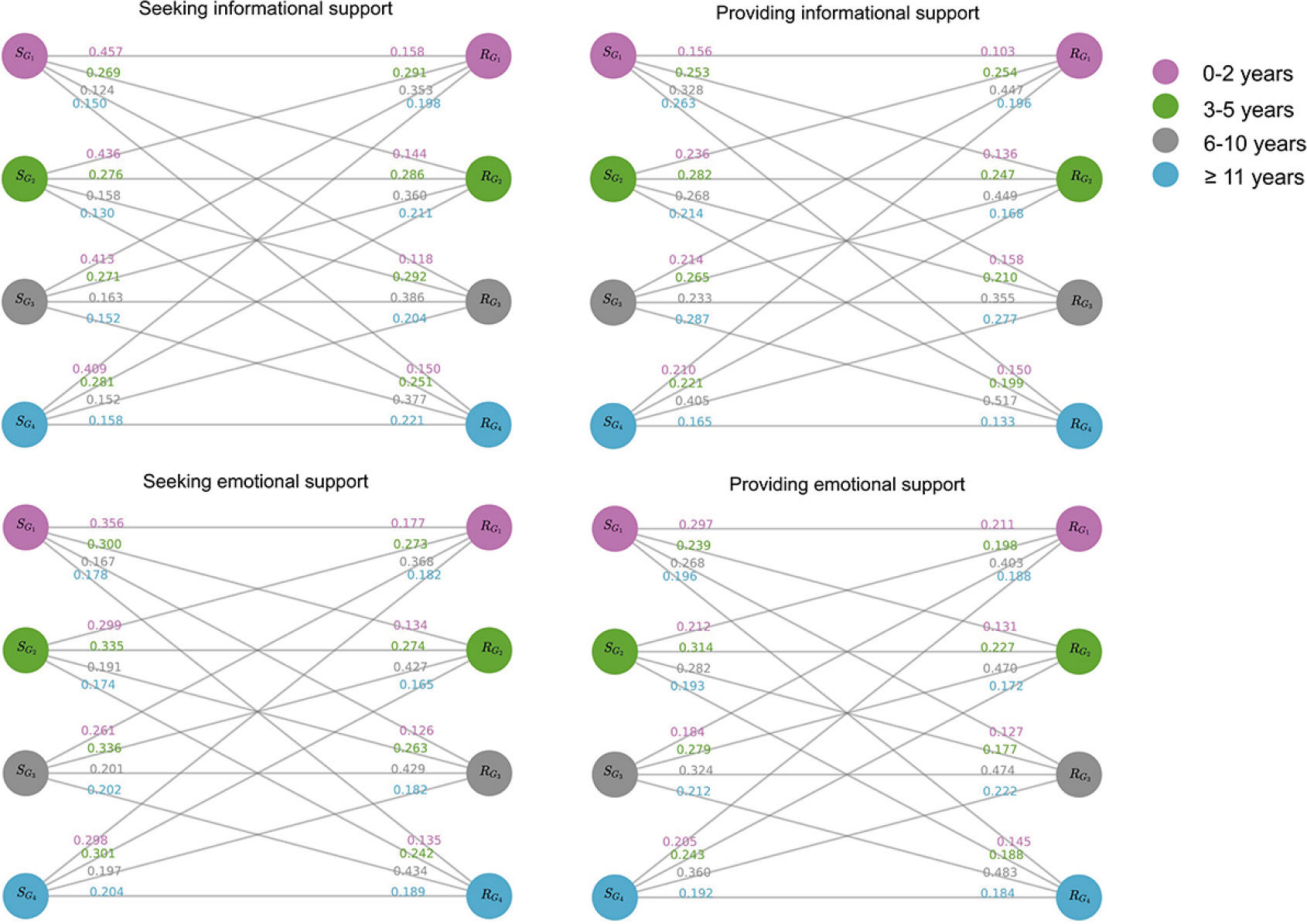
Conditional probability distributions of seeking and providing informational and emotional support across varying disease duration stages.

In social networks, posters typically initiate threads, while commenters engage in follow-up interactions that are often asynchronous and temporally lagged. To avoid interpretive ambiguities in statistical analysis, this study adopts 2 conditional perspectives—one based on the disease duration of posters and the other on that of commenters (refer to Multimedia Appendix 2 for details.) Figure 5 presents the conditional probability distributions of social support provision and seeking across different disease duration groups. In Figure 4, node colors correspond to different stages of disease duration, while node shapes represent different stakeholder identities within the community. Duration groups are denoted as *G*_*i*_ (i=1,2,3,4), with posting users as receive group *R_Gi_*, and commenting users as send group *S_Gi_*.

In the T1D OHC, social support behaviors vary significantly by disease duration. When a stakeholder posts a request for support, individuals in the 0‐2 year group are the least likely to provide either informational or emotional support. For example, in seeking informational and emotional support, the conditional probabilities P(*S_G2_*|*R_G1_*) are 0.158 and 0.177, respectively. As disease duration increases, the probability of stakeholders providing support shows a stepwise upward trend. Those in the 6‐10 year group exhibit the highest willingness to offer support, with probabilities of 0.353 for informational support and 0.368 for emotional support. Among stakeholders with ≥11 years of disease, the probability of providing informational support in response to requests from newly diagnosed users (0‐2 y) P(*R_G1_*|*S*_*G4*_) was 0.409, significantly higher than in other groups (*P*<.001).

Social support interactions exhibited both reciprocity and homophilic mixing based on disease duration. In the informational support dimension, the conditional probability of stakeholders with 6‐10 years of disease duration seeking support when commenting on posts by long-duration users (≥11 y) P(*R_G4_*|*S_G3_*) was 0.287. In contrast, emotional support provision and seeking were largely concentrated within the same disease duration groups. For instance, individuals with 3‐5 years of disease were more likely to interact with peers at the same stage, reflecting a homophilic mixing effect driven by shared illness experiences.

### Regression Analysis

**Figure 6.**
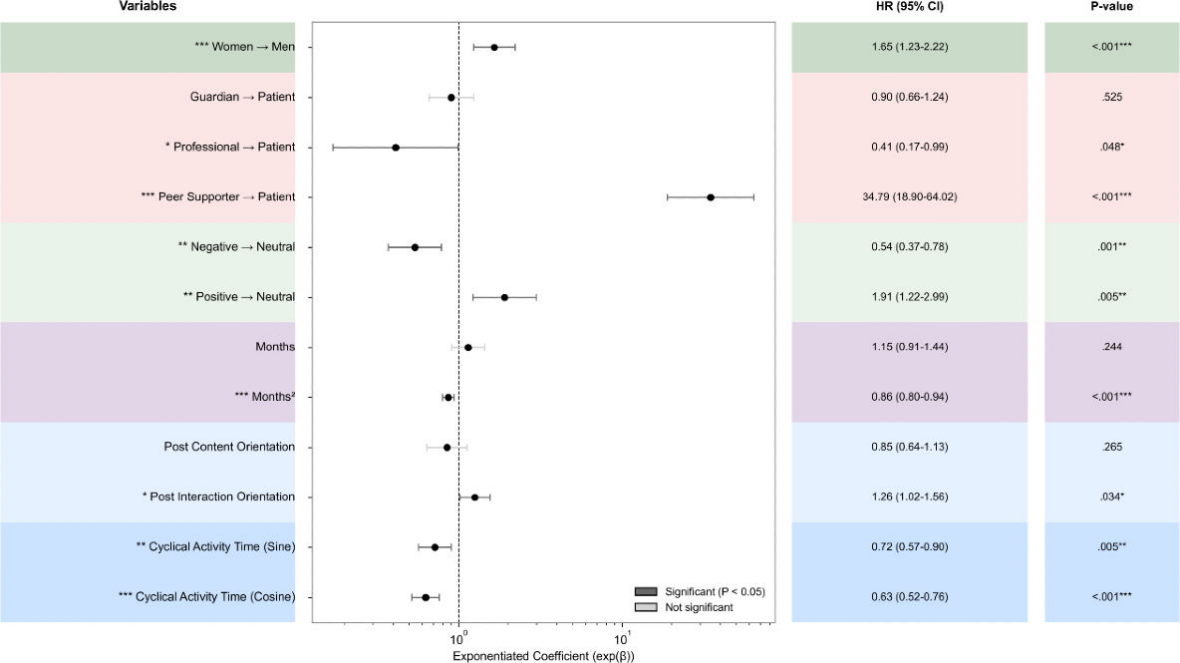
Factors associated with user influence, measured by Relative Centrality, in a social support network. Exponentiated coefficients (exp[β]) from a Gamma-generalized linear model (log link) are presented, with whiskers denoting 95% CIs. *P<.05; **P<.01; ***P<.001.

To further identify which T1D stakeholders exert greater influence within the network and to explore the mechanisms behind it, we developed a behavior-influence analytical framework. This framework links node attributes to network centrality, to examine how high-influence nodes emerge. We estimated a GLM with a gamma distribution and log link, appropriate for the positively skewed, nonnegative distribution of composite relative centrality, which served as the dependent variable. Predictor variables included user demographics, emotional tendencies, posting orientation, cyclical activity patterns, and disease duration. Figure 6 presents exponentiated regression coefficients (exp(β)), interpreted as multiplicative effects on relative centrality. Bars represent estimated coefficients, boxes show SEs, and whiskers denote 95% CIs. Dark gray indicates statistically significant effects.

The results show that both gender and community identity significantly affect user influence within the network. Compared with men, women users demonstrated significantly higher influence (exp(β)=1.65; *P*=.001), indicating approximately 65% greater influence. Among identities, peer supporters exhibited the highest influence, with a relative centrality approximately 34.79 times that of the reference group (patients; exp(β)=34.79; *P*<.001). In contrast, professionals exhibited significantly lower influence, approximately 41% that of patients (exp(β)=0.41; *P*=.055), whereas the influence of guardians did not differ significantly from that of patients (*P*=.53).

Regarding posting orientation, PCO had no significant effect on relative centrality (*P*=.27), indicating that whether a post conveyed informational or emotional content did not affect a user’s network influence. In contrast, PIO was positively associated with centrality (exp(β)=1.26; *P*=.03), suggesting that users who posted with a stronger intent to provide support had, on average, 26% higher influence. This implies that consistently offering support contributes to increased social influence within the community.

In terms of emotional orientation, users expressing more positive emotions exhibited significantly higher centrality, with an increase of 91% compared with those with neutral sentiment (exp(β)=1.91; *P*=.005). Conversely, negative emotional orientation significantly reduced centrality, lowering it to 54% of the neutral group (exp(β)=0.54, *P*=.001). This indicates that positive affect enhances user influence within the T1D community, suggesting that expressions of positivity help elevate one’s position in the support network.

**Figure 7.**
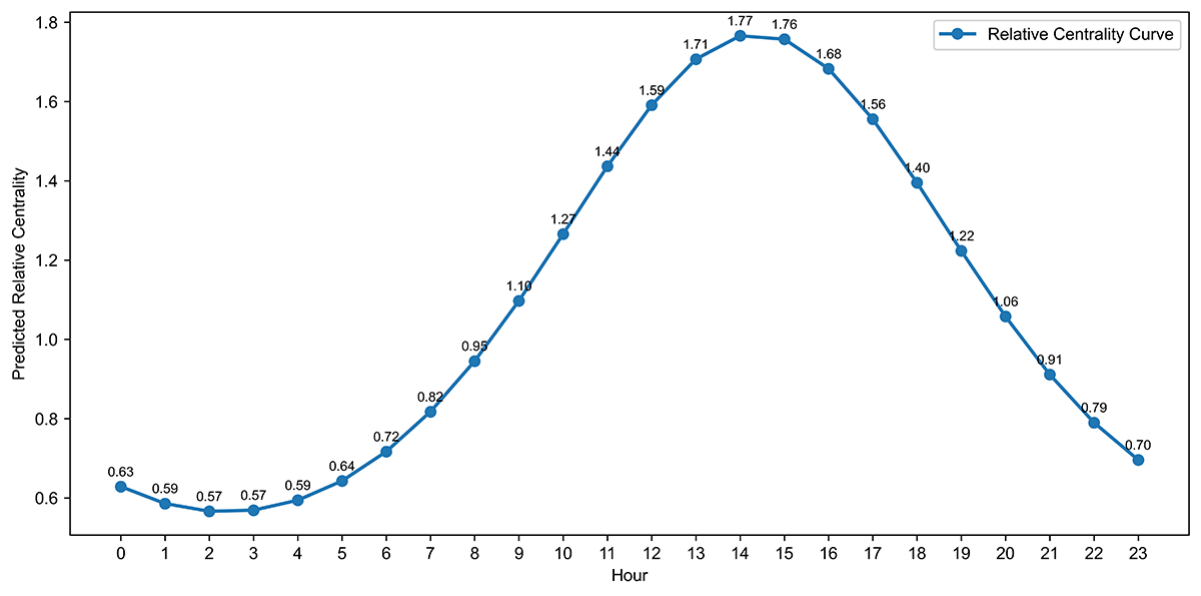
Predicted user influence (Relative Centrality) based on average daily activity time (0‐24 h) among users in China’s largest type 1 diabetes online health community. The curve displays the marginal effect derived from cyclical time-of-day terms (sine and cosine) in a Gamma-generalized linear model (log link).

Additionally, the time of user activity showed a significant association with network influence. Using cyclical activity time (sine/cosine) variables to capture diurnal patterns, we found that both the cosine (exp(β)=0.63; *P*<.001) and sine (exp(β)=0.72; *P*=.005) terms had significant negative effects on relative centrality. This indicates that users active during off-peak hours, such as late at night or early in the morning, tend to have lower influence within the network. To further capture the cyclical effects of posting time, we modeled users’ average active hours based on the estimated coefficients of the sine and cosine terms. The resulting curve (Figure 7) reveals a peak in relative centrality between 2 PM and 3 PM, suggesting that users active during this period tend to hold greater influence in the social support network.

**Figure 8.**
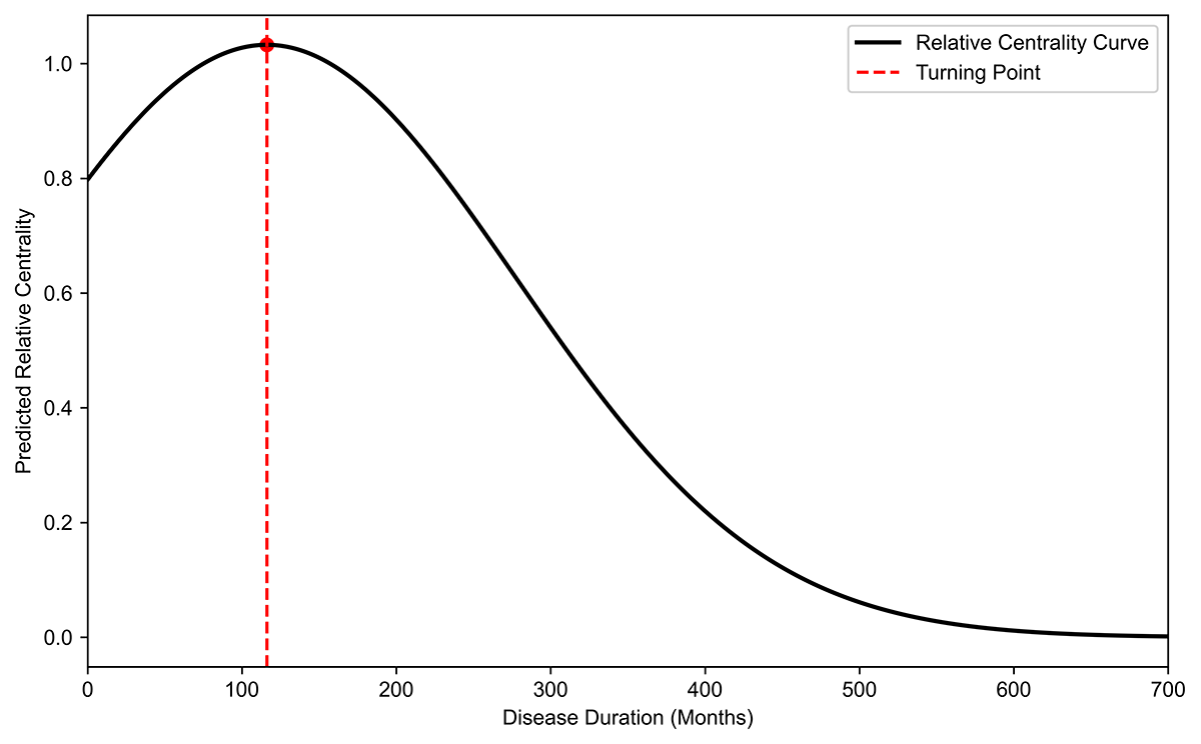
Nonlinear association between disease duration (months since diagnosis or years of service for professionals) and user influence (Relative Centrality), derived from a retrospective observational study of China’s largest type 1 diabetes online health community. The inverted U-shaped marginal effect is calculated from linear and quadratic disease-duration terms in a Gamma-generalized linear model (log link).

In the analysis of disease duration, the quadratic term of months since diagnosis (months^2^) exhibited a significant negative effect on relative centrality (exp(β)=0.87; *P*=.001), while the linear term was not significant (*P*=.24). This suggests an inverted U-shaped relationship between disease duration and user influence, indicating that relative centrality increases in the early stages, peaks, and then declines as disease duration accumulates (Figure 8). Based on the regression coefficients, the peak of relative centrality in the social support network occurs at the 116th month (approximately 9‐10 y) after T1D diagnosis, with a 95% CI of 43.25-188.91, supporting the robustness of the inverted U-shaped pattern [79]. Additionally, the peak influence of patients and peer supporters was observed at 127 months (10‐11 y) and 173 months (14‐15 y), respectively.

### Model Validation and Robustness Check

The model achieved a Cox-Snell pseudo-*R*² of 0.214, indicating a moderate level of model fit. Model diagnostics based on deviance residuals suggested no pronounced systematic deviations. To test the robustness of the observed nonlinear effect, a bootstrap resampling procedure (1200 iterations) was conducted using bias-corrected confidence intervals. The quadratic term for disease duration (months^2^) remained significant (*β*=–.286; *P*=.04), with a 95% CI of –0.545 to −0.013, which did not cross zero. This supports the statistical robustness of the inverted U-shaped relationship between disease duration and relative centrality, independent of normality and homoscedasticity assumptions. In addition, gender, stakeholder identity, and emotional tone also showed significant effects (*P*<.05).

## Discussion

### Principal Findings

This study analyzed the digital footprints from the largest online T1D community in China and applied SNA to construct social support networks involving diverse stakeholders, identifying key influential users and the mechanisms underlying their influence. The findings show that, as disease duration increases, users tend to shift from seeking support to providing support, peer supporters are the most active providers, primarily offering informational support, whereas patients and guardians more frequently seek support and engage in emotional support interactions. Both informational and emotional support subnetworks exhibit multicentric, star-like structures, with the informational subnetwork displaying broader connectivity and greater cross–disease-stage reciprocity, while the emotional subnetwork is smaller, denser, and characterized by homophilous mixing within similar disease stages. Peer supporters occupy the most central network positions, followed by highly active women patients. User influence is significantly associated with positive emotional expression, posting during afternoon time windows, support-oriented content, and disease duration, following a pronounced inverted U-shaped lifecycle trajectory that peaks at approximately 116 months after diagnosis.

Among diverse stakeholders, a clear division of roles emerges within the social support network. Peer supporters function as the most active providers, predominantly offering informational support, whereas patients and guardians primarily act as support seekers and tend to contribute emotional support. Professionals play a complementary role by participating in both informational and emotional support, providing expert guidance alongside peer-based exchanges. Together, these patterns suggest a collaborative and role-differentiated support ecology within the T1D OHC.

From a disease duration perspective, users in the early stages of diagnosis predominantly seek support and gradually transition toward providing support. This pattern aligns with social epidemiological perspectives that emphasize resilience in long-term diabetes management, defined as the capacity to adapt and function positively under chronic stress or prolonged trauma [80]. Existing research indicates that among chronic disease populations such as patients with cardiovascular problems, emotional or self-esteem support is the most significant dimension of social support in predicting quality of life. Its core function lies in enhancing patients’ sense of being valued, cared for, and their psychological coping abilities [81]. In OHCs, informational and emotional support are vital resources for coping with uncertainty and are considered key factors in promoting resilience [82]. As disease experience accumulates, users gradually transition from “seekers” to “providers,” reflecting increasing confidence and competence in disease self-management. This shift not only improves their quality of life [83], but also significantly enhances health literacy and attitudes [84], thereby reinforcing both individual resilience and the collective capacity for collaborative coping within the community.

At the network structure level, the T1D social support network exhibits a complex, multicentered collaborative pattern with cross-disease duration interactions, reflecting a low-centralization topology characteristic of complex networks. Both the subnetworks display star-like topologies with high overlap in node composition, yet significant structural differences exist between them. The informational support subnetwork has a wider coverage and lower density, which facilitates broader user engagement and multisource information diffusion. In contrast, the emotional support subnetwork is more tightly connected, fostering more frequent connections with stable node relationships. These 2 subnetworks jointly reinforce each other, contributing to the connectivity and stability of the online social support network system.

In terms of interaction patterns, informational support follows a reciprocal mechanism across disease durations, whereas emotional support tends to exhibit a homophilic mixing pattern across similar disease durations. Consistent with previous research, the informational support subnetwork is more “instrumental,” with connections spanning multiple duration groups and facilitating knowledge transfer, thereby helping to improve health outcomes and reduce mortality risk [85,86]. The broader, cross-stage pattern of informational exchange reflects a reciprocity mechanism [87], in which users engage in mutual support exchanges across disease stages to seek and transmit practical self-management knowledge. By contrast, emotional support is more “expressive,” relying on strong emotional bonds that enhance users’ sense of community and long-term engagement [86]. The stronger within-stage clustering observed in emotional support exchanges aligns with network homophily, defined as the tendency for individuals to connect with similar others [23]. Moreover, some high-influence stakeholders may gradually evolve into resilience mediators, facilitating emotional adaptation and peer support [82]. Through these complementary informational and emotional exchanges across different disease durations, diverse stakeholders develop a functional division of labor that supports both information flow and emotional connectivity within the system, thereby laying the foundation for the structural resilience and sustainable development of the T1D online social support system.

This study develops a social behavior—node influence framework by systematically linking social support behaviors to network structures and examining how individual attributes and behavioral traits shape relative centrality. In the T1D online social support system, peer supporters serve as the most influential actors while active patients tend to occupy core positions in the network, with influence significantly higher than that of professionals. This highlights the value of the peer “demonstration effect” in chronic disease management, as their continuous participation broadens the scope and depth of information sharing [88]. Compared with professional knowledge, experiential knowledge shared by patients rooted in lived experience and long-term observation of disease progression [26], tends to be more practical and adaptable in managing daily health behaviors and real-life coping. Although professionals, as authoritative information sources guiding health-related discussions and decision-making [89], their structural influence remains relatively limited, likely due to lower participation frequency and narrower interaction scope. This constraint reflects the platform’s operational logic and community culture, which emphasize horizontal, peer-to-peer interactions over hierarchical communication, positioning professionals primarily as supplementary knowledge providers. Guardians occupy a distinct role in family-based care and risk prevention. While their influence does not significantly differ from that of patients, the elevated diabetes risk among patients’ relatives [90,91], highlights the importance of further identifying guardians’ needs and intervention points. This aligns with the current trend of integrating family caregivers into collaborative diabetes management and peer mentoring programs [92]. Furthermore, women demonstrate significantly higher influence than men, possibly due to advantages in metabolic control, emotional expressiveness, and disease self-management [93], thereby facilitating their dominant role in the provision of supportive information.

Beyond individual attributes, influence formation is jointly shaped by microlevel behavioral variables. First, T1D stakeholders who express positive emotions typically exhibit higher relative centrality consistent with evidence that emotional resilience facilitates flexible regulation and engagement in social support interactions [94]. Encouraging positive emotional expression can be an effective strategy to strengthen stakeholder engagement and self-management capabilities. Furthermore, user activity timing also influences their network influence; those active between 2 PM and 3 PM demonstrate markedly higher centrality, reflecting a time-window effect on information visibility and attention clustering [71,72]. Finally, the PCO of posts by T1D stakeholders shows no significant association with their relative centrality, while PIO of posts significantly predicts network position. This aligns with existing research indicating that providing social support enhances health literacy and attitudes more effectively than receiving it [84]. Identifying directional interaction patterns of support providers can help assess their leadership roles in the network, thereby pinpointing credible social influencers [95].

This study further reveals the dynamic trajectory of influence within the T1D online social support system as it evolves over disease duration. The findings indicate that users’ relative centrality follows an inverted U-shaped trajectory correlated with disease duration, peaking at approximately 116 months post diagnosis (10 y), with distinct peaks for patients (127 months) and peer supporters (173 months). In the early stages after diagnosis, as T1D stakeholders gain experience in managing the disease, their influence gradually increases to a peak. However, as the disease progresses, the role of nodes in information brokerage may gradually diminish [57,96]; hyperconnected nodes often become less efficient information conduits due to brokerage overload effects [97]. At the behavioral level, users with longer disease durations often exhibit more established self-management routines and a reduced reliance on community-based informational and emotional support. This may lead to decreased participation frequency and, in some cases, signs of social withdrawal. Moreover, the individualized and long-term experiences they share may not align with the immediate needs of newly diagnosed patients, reducing the contextual relevance and dissemination value of their content. This inverted U-shaped trajectory highlights the dynamic nature of user influence over time and underscores the need for digital health platforms to tailor engagement strategies and knowledge structures according to users’ lifecycle stages.

The findings highlight that identifying and empowering potential social influencers not only enhances community engagement and improves the disease management abilities of other users [98], but also fosters health literacy and behavior change through guided interactions and shared experiences [99]. Based on these findings, it is recommended to further leverage the “demonstration effect” of long-term users within the T1D social network. This would enhance patients’ recognition of the long-term benefits of disease control and strengthen their self-efficacy and autonomy in glycemic management. To operationalize this, the study proposes an innovative patient-centered collaborative care model, led by peer supporters and supported by a multidisciplinary team. The aim is to activate experiential knowledge within the informal caregiving system, optimize information flow, and strengthen emotional connections. In addition, based on the inverted U-shaped lifecycle characteristics of influencers in OHC, it is recommended to establish a mechanism for updating opinion leaders within online communities. This should account for the influence of support behaviors, emotional tendencies, and active time periods on the distribution of influence, thereby continuously refining content design and algorithm-driven content delivery strategies on the platform.

### Limitations

This study has several limitations. First, although the age of onset for T1D is an important factor influencing adult excess mortality [100], the inconsistency and unreliability of age information in user profiles, particularly when entered by caregivers, hinder the accurate reflection of the patient’s age at diagnosis. Additionally, Wu et al [19] found no significant correlation between age at diagnosis and relative centrality, so this variable was excluded from the analysis due to data availability and theoretical relevance. Second, as T1D is an autoimmune disease with heterogeneous characteristics and onset mechanisms [101], this study analyzes disease duration from a general perspective and does not fully capture the individualized disease profiles or specific social support actions of different patients. The impact of social support on health outcomes was also not thoroughly assessed. Future research could integrate longitudinal data and clinical health indicators to explore the role of social support in T1D management more comprehensively. Finally, this study relied solely on text-based data and, due to privacy concerns, did not include multimodal data such as blood glucose monitoring images or videos [48]. Future studies could enhance the robustness and adaptability of classification systems by incorporating diverse data sources and, in conjunction with clinical management data, further investigate the coupling mechanisms between social support network structures and health outcomes.

### Conclusion

In summary, this study elucidates influence formation and social support mechanisms in a large Chinese T1D OHC by integrating stakeholder diversity, disease duration, and network structure. Influence is jointly associated with stakeholder roles, sentiment orientation, activity timing, and supportive interaction patterns. Informational support interactions demonstrate broader participation and stronger cross–disease-stage reciprocity, suggesting a bridging function for knowledge diffusion, whereas emotional support networks are denser and more homophilous, concentrating exchanges among users with similar disease durations. Peer supporters occupy the most central positions, highlighting the critical value of experiential knowledge for maintaining community-based self-management support. Moreover, user influence follows an inverted U-shaped trajectory over the disease duration, implying a lifecycle prominence that platforms may leverage for timely identification and renewal of community facilitators.

This work uses large-scale, real-world digital trace data and integrates disease duration with a multistakeholder perspective, using a reproducible analytic framework that links social support behaviors, network structure, and user influence. These findings may inform the identification and engagement of high-influence peer supporters and the design of disease-stage–tailored community strategies, with professionals involved in targeted complementary roles. Future studies should extend this work with longitudinal and cross-platform data, incorporate comment-level support and content-quality assessment, and link network-based measures to clinical and psychosocial outcomes to clarify how online support relates to health-relevant end points.

## Supplementary material

10.2196/82996 Multimedia Appendix 1 Coding scheme.

10.2196/82996 Multimedia Appendix 2 SentiScore formula and Bayesian conditional probability calculation method.

## Abbreviations

GLM: generalized linear model
LLM: large language model
OHC: online health community
PC: principal component
PCO: postcontent orientation
PIO: postinteraction orientation
RQ: research question
SA: Sentiment analysis
SentiScore: sentiment score
SNA: social network analysis
T1D: type 1 diabetes
