# Supplementary material for: Social Support Mechanisms in an Online Type 1 Diabetes Community: Social Network Analysis of Stakeholder Diversity and Disease Duration
Source: J Med Internet Res. 2026 Jun 15;28:e82996. doi: 10.2196/82996 (PMC13268640; doi:10.2196/82996)
Supplement: Multimedia Appendix 1 [file jmir-v28-e82996-s001.docx]

Coding Scheme

1. Categorization of Social Support Relevance

Posts were categorized as either related or unrelated to social support based on the thematic relevance of their content.

1.1 Unrelated：

The post content is not related to social support.

1.2 Related：

Informational Support:

Involves the exchange of opinions, suggestions, information, or feedback.

Emotional Support:

Involves interactions that express empathy, affection, trust, or care.

1. Definition of Social Support Categories

For posts identified as related to social support, a further categorization was applied to determine whether the post was seeking support or providing support.

2.1 Informational Support

Seeking Informational Support:

The post expresses questions or concerns related to disease management, treatment options, or health strategies, with the intention of receiving opinions, suggestions, information, or feedback.

*e.g., “Hi everyone, my blood glucose levels have been elevated at night for several days. What adjustments should I make? I’d really appreciate your advice.”*

Providing Informational Support:

The post shares knowledge and personal experiences related to disease management, treatment methods, or health strategies. It involves proactively offering opinions, suggestions, information, or feedback.

*e.g., “For two consecutive mornings, I ate a packaged biscuit and had a cup of soy milk powder, then injected 10 units of insulin. My pre- and post-meal blood glucose levels were stable with no upward trend. I'm really happy with the results. I’ve recorded the data, and it seems that 10 units work perfectly when I eat one pack.”*

2.2 Emotional Support

Seeking Emotional Support:

The post conveys emotional distress or negative feelings, with the intention of receiving empathy, affection, trust, or care.

*e.g., “It feels like on this blood sugar control journey, every change along the way has been something I had to figure out on my own. I’m the one learning, making decisions, feeling anxious — no one else. When I don’t get it, the only people I can ask are others with diabetes. Even if I send him a bunch of info, it’s like he doesn’t even see it.”*

Providing Emotional Support:

The post offers empathy, affection, trust, and care, aiming to enhance others’ emotional sense of safety and relieve their emotional distress.

*e.g., “Dear friends with diabetes, I’m so grateful to this world for bringing us together on this journey. We watch over each other, care for each other, and stay connected — sharing warmth, kindness, health, and joy. Wishing all of you good health and stable blood sugar levels in 2024. May the Year of the Dragon bring you peace and blessings.”*
