# Supplementary material for: Social Support Mechanisms in an Online Type 1 Diabetes Community: Social Network Analysis of Stakeholder Diversity and Disease Duration
Source: J Med Internet Res. 2026 Jun 15;28:e82996. doi: 10.2196/82996 (PMC13268640; doi:10.2196/82996)
Supplement: Multimedia Appendix 2 [file jmir-v28-e82996-s002.pdf]

1、SentiScore

The sentiment scores were normalized (SentiScore) to ensure that the sentiment measurements of all samples are comparable on a unified scale.

$$\text{SentiScore} = \begin{cases} P(\text{Positive}), & \text{if } \hat{y} = \text{Positive} \\ 1 - P(\text{Negative}), & \text{if } \hat{y} = \text{Negative} \end{cases}$$

Where $\hat{y}$ represents the predicted label.

2、Bayes' Theorem for Inferring Conditional Probabilities

Let disease duration groups be denoted as G_i,where i=1,2,3,4. The posting users are defined as the Receive Group (R_{G_i}), and the commenting users as the Send Group (S_{G_i}).

$$P(A|B) = \frac{P(B|A)P(A)}{P(B)}$$

For example, if users in the 0–2 year group (G_1) post to seek informational support , and users in the 6–10 year group (G_3) comment on these posts (event X), the posterior probability of event X can be calculated using Bayes' Theorem.

$$P(X) = \mathrm{P}(S_{G_3}|R_{G_1}) = \frac{\mathrm{P}(R_{G_1}|S_{G_3})\,\mathrm{P}(S_{G_3})}{\mathrm{P}(R_{G_1})} = \frac{4.06\%}{11.49\%} = 0.353$$
